# Phenotypic and phylogenetic analyses of Listeria monocytogenes strains reveal enhanced bile tolerance in clinical isolates

**DOI:** 10.1099/jmm.0.002063

**Published:** 2025-09-22

**Authors:** Mary Jane Lynch, Jialun Wu, Olivia McAuliffe, Conor P. O’Byrne, Cormac G.M. Gahan

**Affiliations:** 1 School of Microbiology, University College Cork, Cork, Ireland; 2 Bacterial Stress Response Group, Microbiology, Ryan Institute, School of Biological and Chemical Sciences, University of Galway, Galway, Ireland; 3 Teagasc Food Research Centre, Moorepark, Fermoy, Cork, Ireland; 4 School of Pharmacy, University College Cork, Cork, Ireland; 5 APC Microbiome Ireland, University College Cork, Cork, Ireland

**Keywords:** bile, bile salt hydrolase, gastrointestinal, genomics, *Listeria monocytogenes*, sigma B

## Abstract

**Introduction.** Molecular epidemiological and phenotypic analyses of *Listeria monocytogenes* strains can inform our understanding of factors that influence onward transmission, virulence potential and ability to control the pathogen in foods or in clinical settings. Bile acids represent a host-specific barrier to microbial colonization of the gastrointestinal tract and are, therefore, not encountered in the external environment. We tested the hypothesis that tolerance of bile acids may be an evolutionary adaptation across *L. monocytogenes* clonal complexes (CCs), which varies with genotype and/or is associated with clinical isolates.

**Hypothesis.** We hypothesized that strains of *L. monocytogenes* may differ in bile tolerance (a potential virulence-associated trait) and herein examine this phenomenon according to CC, strain origin and genotype.

**Aim.** To assess 205 genome-sequenced isolates of *L. monocytogenes* for tolerance of porcine bile acids and bile salt hydrolase (BSH) activity.

**Methodology.** Survival of *L. monocytogenes* strains was determined following exposure to porcine bile acids under conditions that mimic the small intestinal environment. BSH activity was assayed against pure taurodeoxycholic acid and glycodeoxycholic acid using an agar plate deconjugation assay. Genomes were analysed for polymorphisms in known bile tolerance loci.

**Results.** Isolates demonstrated distinct inter-individual variances in bile tolerance under anaerobic conditions that mimic the intestinal environment. Strains isolated from cases of human disease were significantly more bile-tolerant than those isolated from natural environments or foods. There was no correlation between levels of bile tolerance and the size of bile precipitation zones on the BSH agar plate assay. No significant patterns were seen upon analysis of known or putative bile tolerance loci; however, individual strains with naturally occurring *sigB* operon mutations demonstrated reduced bile tolerance.

**Conclusion.** *L. monocytogenes* strains isolated from clinical cases of listeriosis demonstrated elevated bile resistance consistent with a likely enhanced capacity to cause gastrointestinal infection preceding invasive disease. The data suggest the potential importance of bile tolerance in *L. monocytogenes* infection and highlight underlying molecular mechanisms by which strains vary in their natural levels of bile tolerance.

## Data Summary

All accession numbers for strains analysed in this study are included in a table as Supplementary Material.

## Introduction

*Listeria monocytogenes* is an important food-borne pathogen that causes severe infection (listeriosis) primarily in immunocompromised individuals. Listeriosis outbreaks have an estimated mortality rate of ~20–30% and the pathogen is a leading cause of death from food-borne illness [1]. *L. monocytogenes* can colonize a wide range of environmental niches, which makes it extremely difficult to eliminate from fresh produce [2]. When contaminated foods are ingested by consumers, the ability to maintain pH homeostasis, potentially through expression of the glutamate decarboxylase system, allows the pathogen to survive the harsh acidic stomach environment and reach the intestine [3,4]. In the small intestine, *L. monocytogenes* can withstand the bile secreted from the gallbladder and subsequently invade intestinal epithelial cells to establish a systemic infection [5]. *L. monocytogenes* can colonize the gallbladder, and this colonization is responsible for the repeated dissemination of *L. monocytogenes* into the gut [6,7]. In some cases, infections are directly associated with the biliary tract, and these correlate with elevated mortality rates [8]. Therefore, the bile stress response of *L. monocytogenes* is of crucial importance to its pathogenic lifestyle.

Bile acids are the main active components of bile, comprising up to 50% of the organic content. They are synthesized from cholesterol in the liver and then solubilized by conjugation (amidation) with glycine or taurine, forming glycodeoxycholic acid (GDCA) and taurodeoxycholic acid (TDCA), respectively [9,10]. Bile is secreted from the liver into the upper intestine, with a significant portion temporarily stored in the gallbladder. It functions as a bio-detergent to emulsify fat in the gut; this is attributed to the amphipathic properties of bile acids. By virtue of these amphipathic properties, bile acids also possess antimicrobial properties, which present as a stress to the gut microbes. Common bile resistance mechanisms include bile salt modification/efflux, cell envelope modification and chaperone/DNA protective effects, suggesting that bile acids exhibit pleiotropic effects on microbes [11,14]. Interestingly, the toxicity of conjugated bile acids is enhanced under anaerobic conditions and at lower pH [15,16]. It was reasoned that protonated bile acids (which are more abundant at lower pH) are more permeable to the cell membrane and thus exert toxic effects similar to those of organic acids.

Bile stress induces a significant change in the transcriptomic and proteomic profile in *L. monocytogenes*, demonstrating the complex nature of bile stress [17,18]. Functional and transcriptomic analyses reveal a significant role for the stress-inducible sigma factor SigB in bile tolerance, primarily through the transcriptional activation of a gene encoding bile salt hydrolase, *bsh* [19,20]. BSH is responsible for the hydrolysis of both GDCA and TDCA and the absence of *bsh* results in bile sensitivity [19,21]. Transcription of *bsh* is also under positive regulation of the master virulence regulator PrfA [19,21]. Interestingly, SigB-dependent transporters OpuC (a carnitine transporter) [22,23] and BilE (a low molecular weight thiol transporter) [24] also contribute to bile tolerance [25,26]. *L. monocytogenes* also exports bile acid using a multidrug efflux pump MdrT [17]. In addition, modifications in the cell envelope also play important roles in bile tolerance [17,27]. Taken together, bile tolerance mechanisms in *L. monocytogenes* are multifaceted and further elucidation is required.

Interestingly, the risks of listeriosis associated with certain *L. monocytogenes* lineages (lineage I) and clonal complexes (CCs) (e.g. CC1, CC2, CC4 and CC6) are disproportionally high compared to their prevalence in food products [28,29]. Little is known about how bile tolerance varies across *L. monocytogenes* wild isolates. As bile resistance is key to gut survival, understanding the strain-to-strain differences in this phenotype can contribute to the overall understanding of why some *L. monocytogenes* strains are more likely to infect humans than others. Recently, we have identified several genetic factors important for growth and survival under food/host-relevant conditions by examining an Irish and European strains collection [30,33]. Herein, we have utilized this existing strain collection and its genome sequences to understand the correlations between bile tolerance and virulence.

A set of 228 *L*. *monocytogenes* isolates of food, environmental and clinical origins [33,34] was phenotypically analysed for bile tolerance and BSH activity. These phenotypes were compared between clinical and non-clinical isolates. Finally, we correlated the phenotypical differences to the genomic variations to pinpoint the potential genetic alterations that account for these phenotypic differences. Overall, the study demonstrated that *L. monocytogenes* isolates of clinical origin exhibit greater levels of bile tolerance than environmental/food isolates, suggestive of an important role in the pathogenesis of individual isolates. Genomic analyses highlighted the importance of individual loci as discussed below.

## Methods

### Bile resistance assay

*L. monocytogenes* cultures were inoculated into brain heart infusion (BHI) broth at pH 7.5 and grown anaerobically (using a gas jar and an anaerobic sachet) or aerobically (shaking) at 37 °C for 18 to 24 h. For the assay, BHI broth (adjusted to pH 5.5 with HCl) was supplemented with 1% dried porcine bile, inoculated 2% with overnight anaerobic/aerobic cultures and incubated at 37 °C for 1 h. Viable plate counts were performed using a BHI broth without bile (T0) and after 1 h (T1) following the addition of bile. Samples were serially diluted in Ringer’s solution and enumerated using BHI agar. Plates were incubated at 37 °C for 18 to 24 h. The Log c.f.u. ml^−1^ was calculated for each sample, and bile resistance was calculated using T0 and T1 differences. Samples were tested in biological and technical triplicates.

### Bile salt hydrolase agar plate assay

Bile salt hydrolase activity was assessed using the plate assay developed by Dashkevicz and Feighner [35]. To prepare the plates, 0.5% (weight/volume) concentrations of bile acids GDCA/TDCA were dissolved in sterile water and added to cooled deMan-Rogosa-Sharpe (MRS) agar as described previously [19]. Plates were stored in an anaerobic jar with an anaerobic sachet for at least 72 h before use. *L. monocytogenes* cultures were grown on BHI agar and patch-inoculated onto the bile acid plates. Plates were incubated anaerobically at 37 °C and read after 48 and 72 h. Positive results for BSH activity present as white zones of precipitation (deconjugated bile acids) surrounding the inoculum. Precipitation zone size diameters were measured. Samples were tested in biological and technical duplicates.

### Genomic analysis

All genome sequences are publicly available at the European Nucleotide Archive under Project accession numbers: PRJEB26051 and PRJEB26050. Methods are adapted from our previous work [33]. Briefly, trimmed pair-end sequencing reads were assembled [36] and annotated using the EGD-e genome as a reference [37]. Resulting genome annotation adapts ‘lmo’ gene nomenclature and facilitates downstream analysis. Phylogeny was inferred by core-genome single-nucleotide polymorphism (SNP) analysis [38], re-rooted at the midpoint. For the examination of polymorphisms among closely related strains, the annotated genome assemblies were aligned using Mauve [39]. To check the conservation across the strains collection for each gene that is known to be involved in bile stress response, the coding sequence was extracted from each genome using a Geneious workflow: ‘For each document’ >> ‘Extract Annotation’ >> ‘Group sequences’ >> ‘Alignment’. The resulting coding sequence alignment was *in silico* translated into protein and checked for amino acid sequence conservation.

### Statistical analysis

All statistical analyses were performed in Prism 10.

## Results

### Bile tolerance profiles of *L. monocytogenes* isolates

The assay conditions for determining bile tolerance of isolates were initially established using six isolates from our collection (Fig. S1, available in the online Supplementary Material). Data confirmed previous studies from our lab [19,20] and suggested that 1-h exposure to 1% porcine bile acids at pH 5.5 under anaerobic conditions significantly reduced *L. monocytogenes* cell numbers in culture media and allowed determination of strain–strain differences in bile tolerance. As previously outlined [19,20], mild acid stress (pH 5.5) was required for effective bile inhibition. The combination of reduced oxygen conditions and mild acid reflects the environment in the upper gastrointestinal tract, where the pathogen first encounters bile acids.

**Fig. 1.**
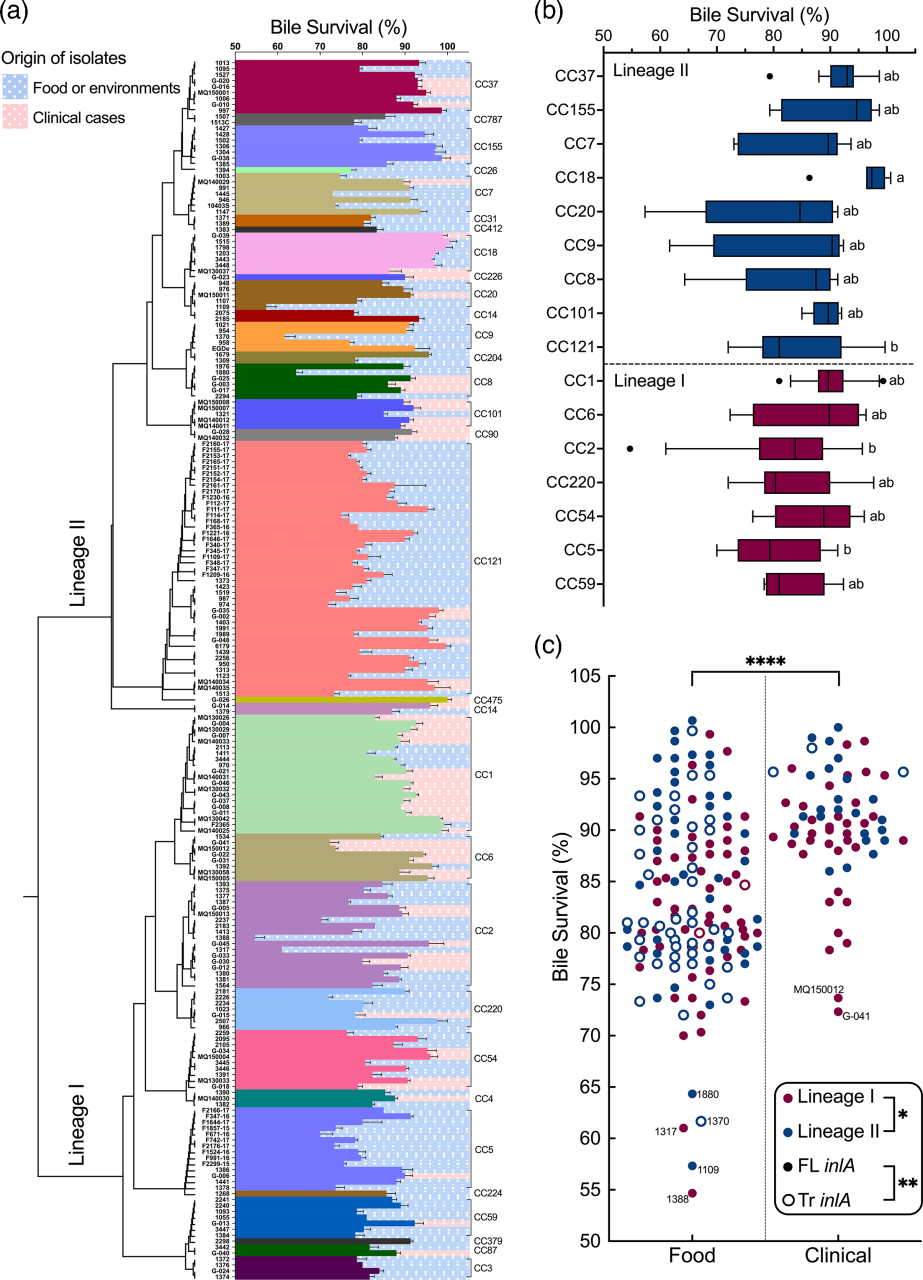
Phylogenetic relatedness of *L. monocytogenes* strains and their phenotypic heterogeneities in bile stress survival. The percentage survival rates of 205 *L*. *monocytogenes* strains following 1-h exposure to bile stress under anaerobic conditions are presented, in conjunction with their phylogenetic relatedness (**a**). In order to analyse the data according to individual CCs, the percentage survival rates of *L. monocytogenes* CCs (with at least five strains) are shown in a box and whiskers (Tukey) plot (**b**). The statistical significance of these differences was determined using the Kruskal–Wallis test, followed by Dunn’s multiple comparisons test (*P*<0.05). In order to assess the data according to strain origin, the percentage survival rates of *L. monocytogenes* strains of food or clinical origin are shown (**c**). The insert shows the results of further statistical comparisons between all lineage I and all lineage II strains and between those strains predicted to express full-length (FL) InlA and those expressing truncated (Tr) InlA. Statistical significances determined for origin, lineages or *inlA* profile in C were calculated using the Kolmogorov–Smirnov test (ns, not significant; *P<0.05; **P<0.01; ***P<0.001; ****P<0.0001).

Phylogenetic clustering of this strain collection was conducted as in a previous study from our group [33], although in the current study, we include a greater number of clinical isolates from cases of listeriosis in Ireland between 2013 and 2020. As expected, isolates clustered into two lineages (lineage I and lineage II) and sub-clustered according to CC type (as determined by *in silico* multilocus sequence typing (MLST) analysis). This permitted analysis of bile tolerance for individual CC types and across lineages (Fig. 1). Considering CCs for which we have at least five representative isolates revealed relatively few differences between CCs with respect to bile tolerance (Fig. 1b). CC18 was significantly more bile tolerant than CC121 and CC5, but we acknowledge that greater sample numbers for individual CC groups will most likely be necessary in order to draw further conclusions.

Direct comparison of bile tolerance in isolates of clinical origin with isolates of food or environmental origin determined that clinical isolates are significantly more tolerant of bile (*n*=205, food/environmental=142, clinical=63) (Fig. 1c). This enhanced tolerance was not evident in strains grown aerobically and challenged in bile under aerobic conditions (*n*=127) (Fig. S2). Comparisons of all lineage I strains with lineage II strains demonstrated a moderate but statistically significant difference between lineages, with lineage II displaying a mean survival measure of 86.3% compared to 84.8% in lineage I isolates (Fig. 1c). We considered whether low bile tolerance may reflect a general phenotypic and genetic transition of strains to a non-pathogenic lifecycle and so examined bile tolerance according to InlA truncation status (Fig. 1c). The analysis indicated that strains encoding full-length InlA display on average a moderately higher measure of bile acid survival in our assay (mean of 86.3%) compared to strains encoding a predicted truncation in InlA (Tr mean of 83.4%).

### BSH activities of *L. monocytogenes* isolates

We determined the optimum conditions for a standard agar plate assay to measure bile acid precipitation following production of the BSH enzyme in *L. monocytogenes* by comparing BHI and MRS agar, porcine bile acids and pure GDCA and TDCA and anaerobic versus aerobic conditions (data not shown). We determined that spot transfer of *L. monocytogenes* colonies onto the surface of MRS plates containing GDCA or TDCA and incubation under anaerobic conditions gave the largest measurable zones of bile acid precipitation. A commercially available mixture of porcine bile acids failed to consistently produce measurable zones of precipitation. The assay was similar to those used previously for *L. monocytogenes* [19] and lactic acid bacteria [40]. The assay permits a qualitative analysis of the BSH activity in hundreds of isolates [40]. We performed the assay to determine whether all isolates produce detectable BSH activity and whether there is a correlation between size of zones of precipitation and bile tolerance of strains.

**Fig. 2.**
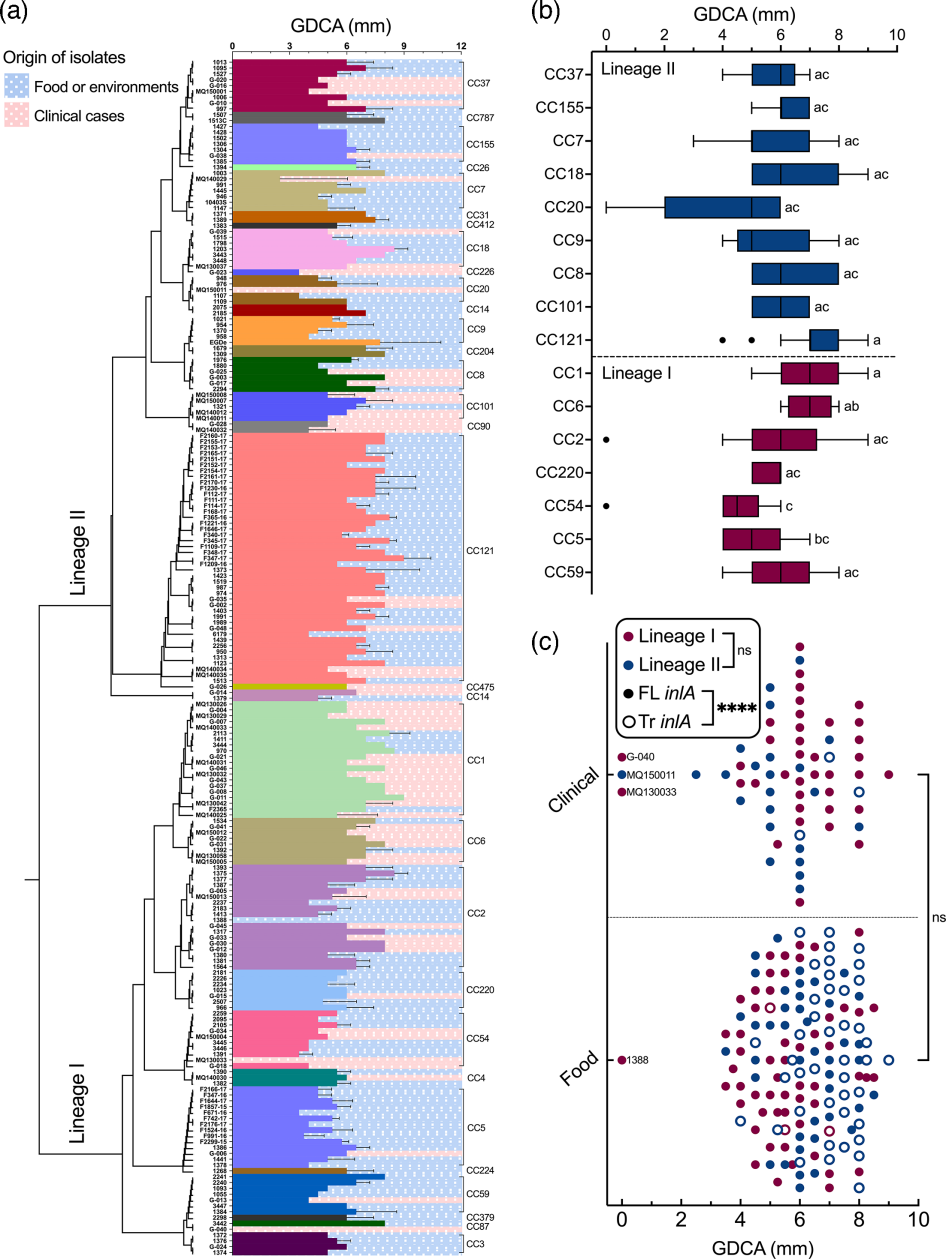
Phylogenetic relatedness of *L. monocytogenes* strains and their phenotypic heterogeneities in GDCA activity. The GDCA activities of 205 *L*. *monocytogenes* strains are presented as the diameter (in mm) of the zone of precipitation, in conjunction with their phylogenetic relatedness (**a**). The GDCA activities of *L. monocytogenes* CCs (with at least five strains) are shown in a box and whiskers (Tukey) plot (**b**). The statistical significance of these differences was determined using the Kruskal–Wallis test, followed by Dunn’s multiple comparisons test (*P*<0.05). The percentage survival rates of *L. monocytogenes* strains of food or clinical origin are shown (**c**). Statistical significances were determined for origin, lineages or *inlA* profile (FL, full length; Tr, truncation), using the Kolmogorov–Smirnov test (ns, not significant; *P<0.05; **P<0.01; ***P<0.001; ****P<0.0001).

Analysis of strains for GDCA deconjugation activities according to phylogeny indicated variation of zone sizes (Fig. 2a). Statistical analysis revealed that CC121, CC1 and CC6 displayed larger GDCA zones than CC54 (Fig. 2b). However, again, greater numbers of isolates in individual CCs may be required to draw definitive conclusions. We note that four isolates (clinical isolates MQ150011, MQ130033, G-040 and food isolate 1388) failed to produce zones of precipitation on the GDCA agar plate assay. This negative BSH phenotype did not correlate with bile tolerance of these four isolates. Strain 1388 had the lowest % bile resistance (55%) of all strains tested, but the three clinical strains MQ130033, MQ150011 and G40 had relatively high levels of bile resistance (91%, 91% and 88%, respectively). Zones of bile acid precipitation on TDCA agar plates were smaller than those on GDCA agar plates and greater numbers of isolates failed to produce zones on TDCA (Fig. S3). Interestingly, the well-studied laboratory strain *L. monocytogenes* EGDe produced zones of precipitation on both GDCA and TDCA agar plates. A correlation between GDCA and TDCA activities was demonstrated (Fig. S4). For instance, the four isolates that tested negative for activity on GDCA were also negative for activity on TDCA, whilst particular strains (e.g. 997, F347-17, EGDe and 1373) which produced larger zones on GDCA plates also produced demonstrable TDCA activity. We postulate that the TDCA assay has a higher limit of detection than the GDCA assay and, as reported in other studies, suggest a preference for the use of GDCA in BSH assays for this species [19,20].

We determined that there was no significant correlation between BSH activity on the GDCA agar plate assay (Fig. 2) and *in vitro* bile tolerance on porcine bile (Fig. 1) (see Fig. S5). For instance, the clinical isolates that were negative for BSH activity in our assays (MQ150011, MQ130033 and G-040) demonstrated relatively high levels of bile tolerance (Fig. 2). This suggests that microbial systems other than BSH may be important for overall bile tolerance or that *in vivo* factors/conditions that we have not included in our assay are important for *bsh* expression in the gastrointestinal (GI) tract. Studies of BSH activity in other species have indicated that whilst the plate assay determines the existence of BSH activity in a species, it was developed as a qualitative rather than quantitative assay [40]. Future studies are necessary to precisely quantify BSH activities across strains and to analyse the importance of other genetic systems for bile tolerance in *L. monocytogenes*.

### Genomic analyses

To determine whether bile tolerance and BSH activities among this strain collection reflect genetic differences between strains, we examined the conservation of amino acid sequences of known bile stress response determinants among 205 genomes (Table 1 represents analysis of lineage I strains, whereas lineage II strains are presented in Table 2). Substitutions, truncations, insertions or deletions are described in these loci, and these are correlated with the strains that display different phenotypes compared with closely related strains (of the same CC). For this analysis, the polymorphisms that are strictly associated with lineages were excluded. This analysis highlighted the involvement of *sigB* operon mutations in strains 1388 and 1370 with bile tolerance and GDCA activity. In addition, the BrtA L226I mutation was only evident in a bile-sensitive CC2 (strain 1317). Despite the known contribution of BrtA to bile tolerance [41], the conservative nature of this substitution is unlikely to drastically alter BrtA function. No other substitution in the genes examined was associated with bile tolerance or BSH activity. These results further highlight the critical role of SigB in the bile stress response as well as the complex, multifactorial nature of the bile stress response.

**Table 1.** Amino acid substitutions in bile stress-related genes within lineage I

**Table 2.** Amino acid substitutions in bile stress-related genes within lineage II

Interestingly, CC20 strains 1107 and 1109 display differences in bile tolerance despite high genome identity (differ by only four loci). Of note among these is a frameshift mutation of *clsA*/*lmo2503* found in strain 1109 (Table S1, available in the online version of this article). *clsA* encodes a putative cardiolipin synthetase, and this frameshift results in a truncated translation product of 448 aa, whilst strain 1107 and other strains in this study encode full-length ClsA (483 aa). Cardiolipin has been implicated in bile tolerance in *L. monocytogenes* [42] and other bacteria [12,43]. Evidence suggests that cardiolipin increases the resistance of phosphatidylglycerol membranes to solubilization by bile acids [12]. Given that the other three polymorphisms are unlikely to influence bile tolerance, the likely disrupted cardiolipin synthesis plausibly explains the bile-sensitive phenotype of strain 1109.

## Discussion

Phenotypic analysis of *L. monocytogenes* isolates is necessary to delineate traits associated with particular CCs or lineages and to determine features of clinical isolates that may favour infection. Here, we investigated the bile tolerance of 205 individual isolates of *L. monocytogenes* from both food/environmental and clinical sources. We show significant variation in bile tolerance across individual isolates. Under conditions that mimic the small intestine (anaerobicity and low pH), clinical isolates were significantly more bile-tolerant than environmental/food isolates. The results suggest an adaptation of *L. monocytogenes* strains to conditions encountered in the small intestine, which may favour an infectious lifecycle or onward transmission of the pathogen.

Bile acids represent a host-restricted sub-optimal stressor that foodborne pathogens encounter in the GI tract. We have previously shown that *L. monocytogenes* survival is impeded by bile acids under mildly acidic conditions (pH 5.5), such as those encountered in the small intestine [20]. In contrast, *ex vivo* bile acids from the gall bladder at neutral pH do not impede the growth or survival of the pathogen [20]. Indeed, the gall bladder appears to represent a favourable niche for the pathogen, which is capable of significant growth in the gall bladder of mice [6]. Therefore, we wished to determine the survival of *L. monocytogenes* isolates under conditions encountered in the small intestine. An unbiased analysis revealed significantly higher levels of bile tolerance associated with strains of clinical origin, provided insights into *in vitro* BSH activities across strains and permitted genomic analyses for potential mechanisms. Whilst CC18 demonstrated elevated bile tolerance in our study, we did not see significant differences between other CCs and suggest that higher numbers of isolates across different CCs will be necessary to determine whether such differences exist.

Previous work has shown that the BSH enzyme of *L. monocytogenes* contributes to survival in bile acids [19,21], notably under conditions that mimic the small intestine [19,20]. The *bsh* gene is regulated by both the stress regulator SigmaB and the virulence regulator PrfA, and a *bsh* mutant shows diminished survival in the GI tract of mice and during systemic infection [19,21]. Indeed, the non-pathogenic species *Listeria innocua* naturally lacks the *bsh* gene [19,21], and re-introduction of *bsh* into *L. innocua* significantly improves the ability to survive bile acids and to colonise the GI tract of mice [44]. In the current study, the majority of isolates produced zones of precipitation (indicative of BSH activity) against the bile acid GDCA in an agar plate assay, though zone size did not correlate with overall bile resistance, suggesting that other loci are also important for bile resistance or that the induction of *bsh* expression depends upon conditions/factors not represented in our assay.

Indeed, other loci responsible for bile tolerance in *L. monocytogenes* have been described. These include *mdrT*, which encodes a putative bile acid transporter; *opuC*, which encodes a carnitine transporter; and *bilE*, which encodes a thiol transporter (with a proposed revised gene designation of *egtU* (originally genes *lmo1421* and *lmo1422* in strain EGDe)) [24,45]. We performed genomic analyses in our strain collection to identify non-canonical amino acids or truncations in these loci as well as genes encoding the relevant transcriptional regulators BrtA, SigB and PrfA. Notably, one strain (1388) (previously described in [33]) carried a truncation in the gene encoding SigB and demonstrated reduced bile tolerance and did not express BSH activity in our assays. The genome of environmental isolate 1370 contains a truncation in the gene encoding the anti-anti-sigma factor RsbV, which positively regulates SigB activity, and also demonstrates greatly reduced bile tolerance. A clinical isolate, MQ140025, exhibited heightened bile tolerance but carries a truncation in the RsbU locus, which encodes a diphosphatase and would be expected to impair SigB activity [33]. The reasons for this phenotype are, therefore, unclear but may reflect compensatory expression of other bile tolerance loci and could form the basis of further study.

In conclusion, we have shown that clinical isolates of *L. monocytogenes* are more likely than environmental isolates to demonstrate elevated bile tolerance. Given that the pathogen encounters bile acids early in the infectious cycle (within the small intestine), it is likely that this enhances the ability to cause infection, though we appreciate that many other factors also play a role.

## Supplementary material

10.1099/jmm.0.002063 Uncited Supplementary Material 1.

## Abbreviations

BHI: brain heart infusion
BSH: bile salt hydrolase
CCs: clonal complexes
GDCA: glycodeoxycholic acid
GI: gastrointestinal
MRS: deMan-Rogosa-Sharpe
TDCA: taurodeoxycholic acid
