# Supplementary material for: Phenotypic and phylogenetic analyses of Listeria monocytogenes strains reveal enhanced bile tolerance in clinical isolates
Source: J Med Microbiol. 2025 Sep 22;74(9):002063. doi: 10.1099/jmm.0.002063 (PMC12476152; doi:10.1099/jmm.0.002063)
Supplement: Uncited Supplementary Material 1. [file jmm-74-02063-s001.pdf]

*Supplementary Material*

**Lynch et al. 2025. Phenotypic and phylogenetic analyses of *Listeria monocytogenes* strains reveal enhanced bile tolerance in clinical isolates.**

**Fig. S1.**

**(a)**

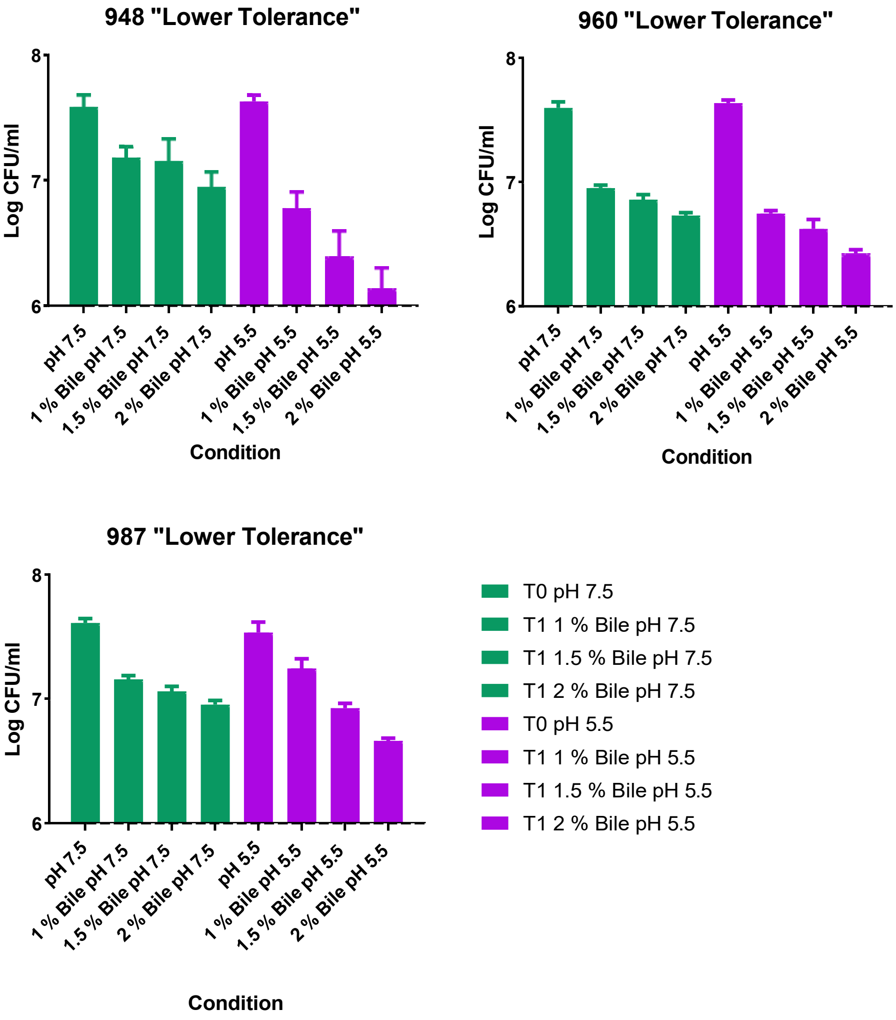

**(b)**

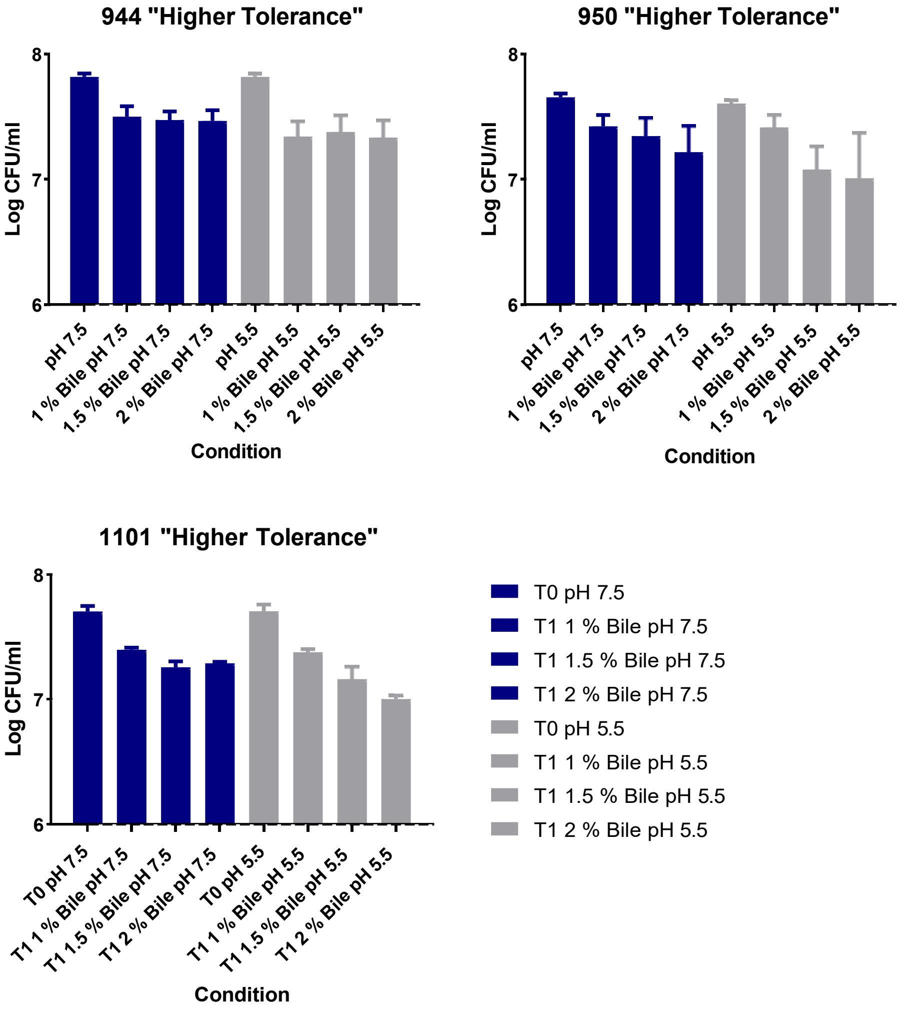
944 "Higher Tolerance"
950 "Higher Tolerance"
1101 "Higher Tolerance"
Log CFU/ml
8
7
6
pH 7.5
1 % Bile pH 7.5
1.5 % Bile pH 7.5
2 % Bile pH 7.5
pH 5.5
1 % Bile pH 5.5
1.5 % Bile pH 5.5
2 % Bile pH 5.5
T0 pH 7.5
T1 1 % Bile pH 7.5
T1 1.5 % Bile pH 7.5
T1 2 % Bile pH 7.5
T0 pH 5.5
T1 1 % Bile pH 5.5
T1 1.5 % Bile pH 5.5
T1 2 % Bile pH 5.5
Condition

**(c)**

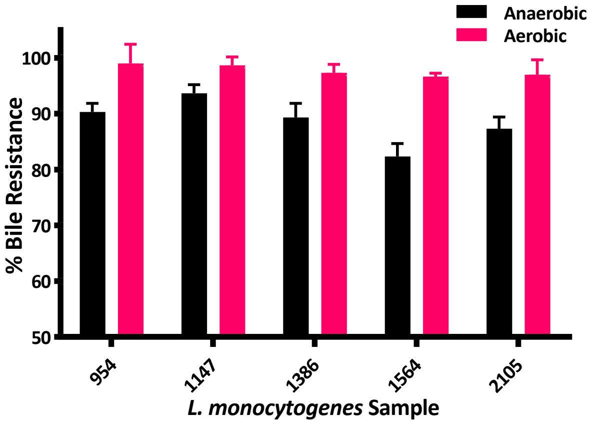
Anaerobic
Aerobic
% Bile Resistance
100
90
80
70
60
50
954
1147
1386
1564
2105
*L. monocytogenes* Sample

**Figure S1.** Effect of bile concentration and pH on bile tolerance profiles. Preliminary results (not shown) permitted the selection of **(a)** three strains exhibiting a 'lower tolerance' to bile (Figure S1A: 948, 960, 987) and three strains **(b)** exhibiting a 'higher tolerance' to bile (Figure S1B: 944, 950, 1101) were selected for testing.  Different bile concentrations (1, 1.5 and 2% porcine bile) and pH conditions (pH7.5 and pH5.5) were investigated. **(c)** Effect of atmospheric conditions on bile resistance. Cultures were grown aerobically or anaerobically for 18 hours before inoculating into a BHI broth/BHI bile broth. In all cases samples were serially diluted in Ringer's solution and enumerated using BHI agar. Plates were incubated at 37 °C for 24 hours. The Log CFU/ml was calculated for each sample. Comparison of the Log CFU/ml for the T0 value with the T1/T2 bile broth values demonstrated the effect of bile on the different conditions trialled. Samples were tested in biological and technical triplicates. The error bars represent the mean +/- standard deviation.

**Fig. S2**

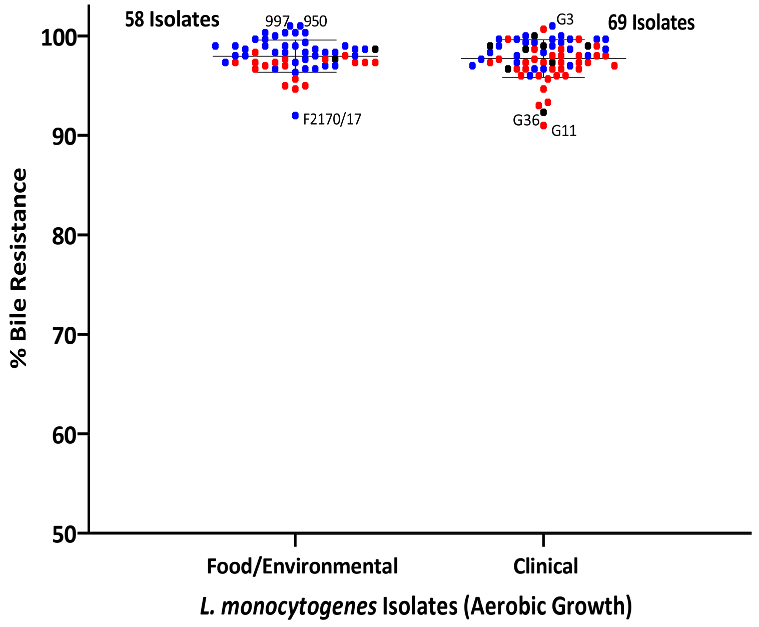


**Fig. S2.** Bile resistance results for aerobically grown isolates grouped by lineage and sample origin (food/environmental 58 isolates, clinical 69 isolates). Red represents lineage I strains and blue represents lineage II strains. Black represents strains with unknown lineage. *L. monocytogenes* cultures were grown aerobically in BHI broth for 18 to 24 hours. For the assay, BHI broth (pH 5.5) was supplemented with 1% dried porcine bile and inoculated 2% with overnight cultures. Viable plate counts were performed using a BHI broth without bile (Time 0) and after one hour (Time 1) for the BHI bile broth. Samples were serial diluted in Ringer's solution and enumerated using BHI agar. Bile resistance was calculated using T0 and T1 differences. Samples were tested in biological and technical triplicates. The error bars represent the mean +/- standard deviation. There was no significant difference between groups.

**Fig. S3.** BSH activity of strains on TDCA plates. Diameter of zones of precipitation according to phylogeny.

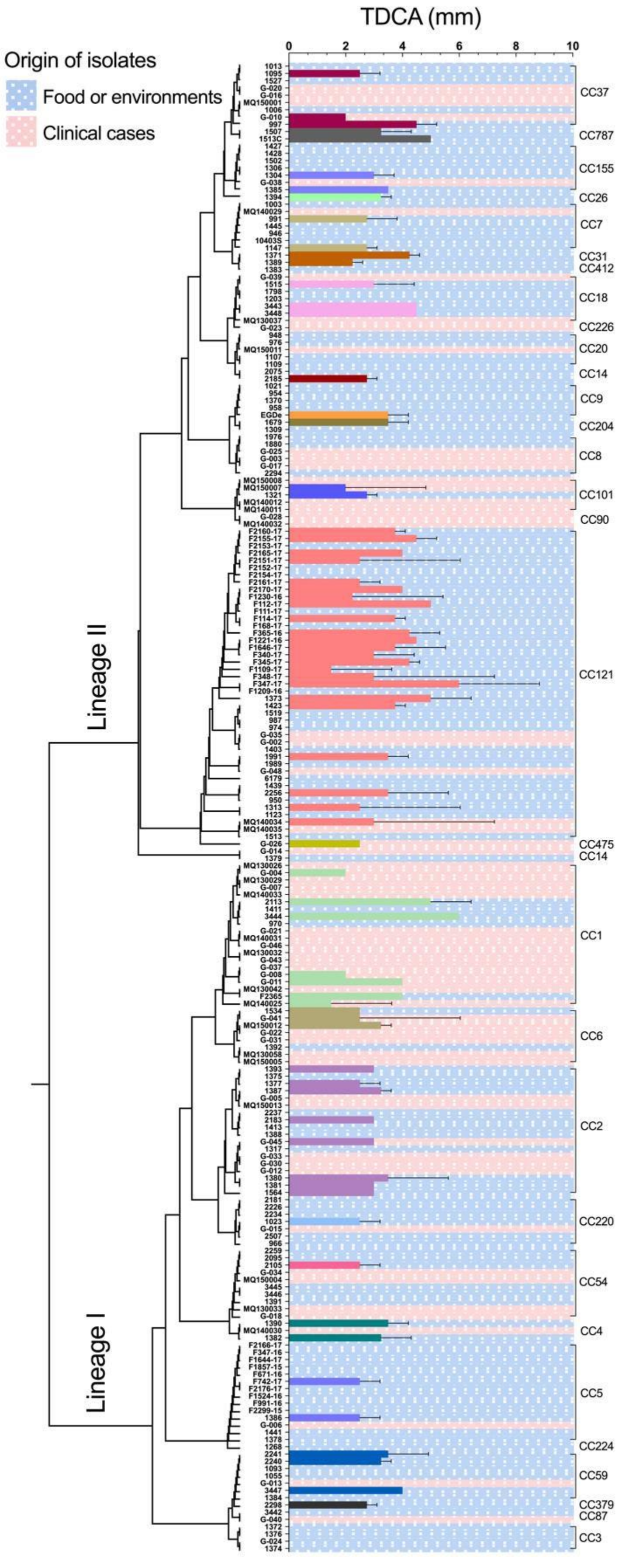

**Fig. S4.** Pearson correlation comparing TDCA and GDCA activities of strains.

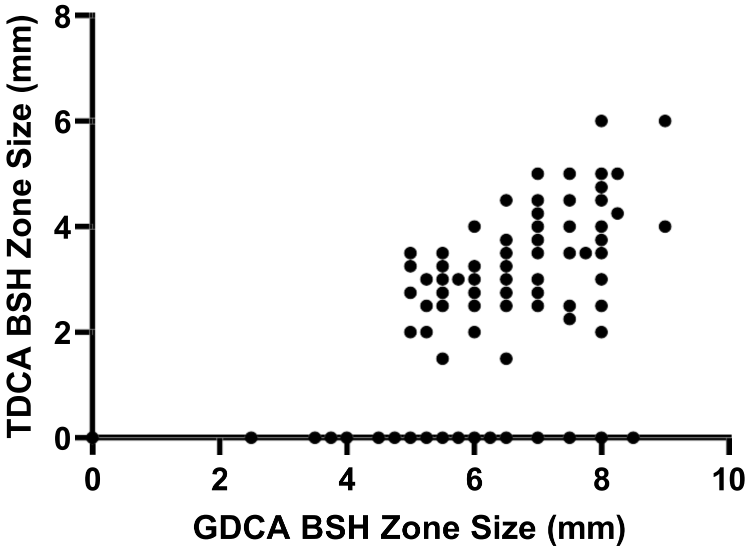


| | |
|---|---|
| **Pearson r** | |
| r | 0.3922 |
| 95% confidence interval | 0.2764 to 0.4968 |
| R squared | 0.1538 |
| P value | |
| P (two-tailed) | <0.0001 |
| P value summary | **** |
| Significant? (alpha = 0.05) | Yes |
| Number of XY Pairs | 228 |

**Fig. S5.** Pearson correlation comparing GDCA activities of strains with bile tolerance.

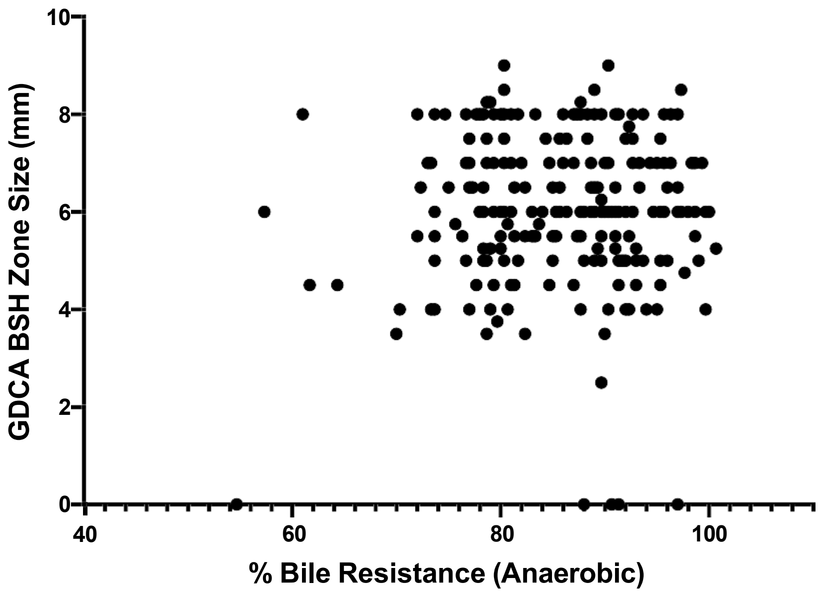


| | |
|---|---|
| **Pearson r** | |
| r | 0.01996 |
| 95% confidence interval | -0.1103 to 0.1495 |
| R squared | 0.0003982 |
| P value | |
| P (two-tailed) | 0.7644 |
| P value summary | ns |
| Significant? (alpha = 0.05) | No |
| Number of XY Pairs | 228 |
| **Spearman r** | |
| r | -0.02473 |
| 95% confidence interval | -0.1579 to 0.1094 |
| P value | |
| P (two-tailed) | 0.7103 |
| P value summary | ns |
| Exact or approximate P value? | Approximate |
| Significant? (alpha = 0.05) | No |
| Number of XY Pairs | 228 |

**Table of Genome-sequenced *L. monocytogenes* strains used in this study with Accession Numbers**

| Strain name | Source | Database | Bioproject | Accession | Reference |
|---|---|---|---|---|---|
| 946 | Environment | NCBI | PRJNA796187 | SAMN24821055 | Wu et al., 2022 |
| 948 | Environment | NCBI | PRJNA796187 | SAMN24821056 | Wu et al., 2022 |
| 950 | Environment | NCBI | PRJNA796187 | SAMN24821057 | Wu et al., 2022 |
| 954 | Environment | NCBI | PRJNA796187 | SAMN24821058 | Wu et al., 2022 |
| 958 | Environment | NCBI | PRJNA796187 | SAMN24821059 | Wu et al., 2022 |
| 966 | Environment | NCBI | PRJNA699172 | JAFDOA000000000 | Wu et al., 2022 |
| 970 | Environment | NCBI | PRJNA796187 | SAMN24821060 | Wu et al., 2022 |
| 974 | Environment | NCBI | PRJNA699172 | JAFDNZ000000000 | Wu et al., 2022 |
| 976 | Mixed foods | NCBI | PRJNA699172 | JAFDNY000000000 | Wu et al., 2022 |
| 987 | Environment | NCBI | PRJNA796187 | SAMN24821061 | Wu et al., 2022 |
| 991 | Meat | NCBI | PRJNA796187 | SAMN24821062 | Wu et al., 2022 |
| 997 | Environment | NCBI | PRJNA796187 | SAMN24821063 | Wu et al., 2022 |
| 1003 | Meat | NCBI | PRJNA796187 | SAMN24821064 | Wu et al., 2022 |
| 1006 | Environment | NCBI | PRJNA796187 | SAMN24821065 | Wu et al., 2022 |
| 1013 | Environment | NCBI | PRJNA796187 | SAMN24821066 | Wu et al., 2022 |
| 1021 | Environment | NCBI | PRJNA796187 | SAMN24821067 | Wu et al., 2022 |
| 1023 | Dairy | NCBI | PRJNA796187 | SAMN24821068 | Wu et al., 2022 |
| 1055 | Environment | NCBI | PRJNA699172 | JAFDNX000000000 | Wu et al., 2022 |
| 1093 | Environment | NCBI | PRJNA699172 | JAFDNW000000000 | Wu et al., 2022 |
| 1095 | Environment | NCBI | PRJNA699172 | JAFDNV000000000 | Wu et al., 2022 |
| 1107 | Environment | NCBI | PRJNA796187 | SAMN24821069 | Wu et al., 2022 |
| 1109 | Environment | NCBI | PRJNA796187 | SAMN24821070 | Wu et al., 2022 |
| 1123 | Seafood | NCBI | PRJNA699172 | JAFDNU000000000 | Wu et al., 2022 |

| | | | | | |
|---|---|---|---|---|---|
| 1147 | Environment | NCBI | PRJNA796187 | SAMN24821071 | Wu et al., 2022 |
| 1203 | Environment | NCBI | PRJNA699172 | JAFDNT000000000 | Wu et al., 2022 |
| 1268 | Dairy | NCBI | PRJNA699172 | JAFDNS000000000 | Wu et al., 2022 |
| 1304 | Environment | NCBI | PRJNA698557 | JAFFOC000000000 | Wu et al., 2022 |
| 1306 | Environment | NCBI | PRJNA698557 | JAFFOD000000000 | Wu et al., 2022 |
| 1309 | Environment | NCBI | PRJNA698557 | JAFFOE000000000 | Wu et al., 2022 |
| 1313 | Environment | NCBI | PRJNA698557 | JAFFOF000000000 | Wu et al., 2022 |
| 1317 | Environment | NCBI | PRJNA698557 | JAFFOG000000000 | Wu et al., 2022 |
| 1321 | Environment | NCBI | PRJNA698557 | JAFFOH000000000 | Wu et al., 2022 |
| 1370 | Meat | NCBI | PRJNA808240 | JAKTOT000000000 | Wu et al., 2022 |
| 1371 | Meat | NCBI | PRJNA808240 | JAKTOS000000000 | Wu et al., 2022 |
| 1372 | Meat | NCBI | PRJNA808240 | JAKTOR000000000 | Wu et al., 2022 |
| 1373 | Meat | NCBI | PRJNA808240 | JAKTOQ000000000 | Wu et al., 2022 |
| 1374 | Meat | NCBI | PRJNA808240 | JAKTOP000000000 | Wu et al., 2022 |
| 1375 | Meat | NCBI | PRJNA808240 | JAKTOO000000000 | Wu et al., 2022 |
| 1376 | Meat | NCBI | PRJNA808240 | JAKTON000000000 | Wu et al., 2022 |
| 1377 | Meat | NCBI | PRJNA808240 | JAKTOM000000000 | Wu et al., 2022 |
| 1378 | Dairy | NCBI | PRJNA808240 | JAKTOL000000000 | Wu et al., 2022 |
| 1379 | Dairy | NCBI | PRJNA808240 | JAKTOK000000000 | Wu et al., 2022 |
| 1380 | Meat | NCBI | PRJNA808240 | JAKTOJ000000000 | Wu et al., 2022 |
| 1381 | Meat | NCBI | PRJNA808240 | JAKTOI000000000 | Wu et al., 2022 |
| 1382 | Dairy | NCBI | PRJNA808240 | JAKTOH000000000 | Wu et al., 2022 |
| 1383 | Dairy | NCBI | PRJNA808240 | JAKTOG000000000 | Wu et al., 2022 |
| 1384 | Seafood | NCBI | PRJNA808240 | JAKTOF000000000 | Wu et al., 2022 |
| 1385 | Seafood | NCBI | PRJNA808240 | JAKTOE000000000 | Wu et al., 2022 |

| | | | | | |
|---|---|---|---|---|---|
| 1386 | Seafood | NCBI | PRJNA808240 | JAKTOD000000000 | Wu et al., 2022 |
| 1387 | Seafood | NCBI | PRJNA808240 | JAKTOC000000000 | Wu et al., 2022 |
| 1388 | Seafood | NCBI | PRJNA808240 | JAKTOV000000000 | Wu et al., 2022 |
| 1389 | Seafood | NCBI | PRJNA808240 | JAKTOB000000000 | Wu et al., 2022 |
| 1390 | Dairy | NCBI | PRJNA808240 | JAKTOA000000000 | Wu et al., 2022 |
| 1391 | Dairy | NCBI | PRJNA808240 | JAKTNZ000000000 | Wu et al., 2022 |
| 1392 | Seafood | NCBI | PRJNA808240 | JAKTNY000000000 | Wu et al., 2022 |
| 1393 | Meat | NCBI | PRJNA808240 | JAKTOU000000000 | Wu et al., 2022 |
| 1394 | Dairy | NCBI | PRJNA808240 | JAKTNX000000000 | Wu et al., 2022 |
| 1403 | Seafood | NCBI | PRJNA698557 | JAFFOI000000000 | Wu et al., 2022 |
| 1411 | Environment | NCBI | PRJNA699172 | JAFDNR000000000 | Wu et al., 2022 |
| 1413 | Environment | NCBI | PRJNA699172 | JAFDNQ000000000 | Wu et al., 2022 |
| 1423 | Environment | NCBI | PRJNA699172 | JAFDNP000000000 | Wu et al., 2022 |
| 1427 | Environment | NCBI | PRJNA698557 | JAFFOJ000000000 | Wu et al., 2022 |
| 1428 | Environment | NCBI | PRJNA698557 | JAFFOQ000000000 | Wu et al., 2022 |
| 1439 | Smoked Salmon | NCBI | PRJNA699172 | JAFDNO000000000 | Wu et al., 2022 |
| 1441 | Environment | NCBI | PRJNA699172 | JAFDNN000000000 | Wu et al., 2022 |
| 1445 | Meat | NCBI | PRJNA699172 | JAFDNM000000000 | Wu et al., 2022 |
| 1502 | Environment | NCBI | PRJNA698557 | JAFFOK000000000 | Wu et al., 2022 |
| 1507 | Environment | NCBI | PRJNA698557 | JAFFOL000000000 | Wu et al., 2022 |
| 1513 | Seafood | NCBI | PRJNA699172 | JAFDNL000000000 | Wu et al., 2022 |
| 1515 | Environment | NCBI | PRJNA698557 | JAFFON000000000 | Wu et al., 2022 |
| 1519 | Seafood | NCBI | PRJNA698557 | JAFFOO000000000 | Wu et al., 2022 |
| 1527 | Environment | NCBI | PRJNA698557 | JAFFOP000000000 | Wu et al., 2022 |
| 1534 | Meat | NCBI | PRJNA699172 | JAFDNK000000000 | Wu et al., 2022 |

| | | | | | |
|---|---|---|---|---|---|
| 1564 | Environment | NCBI | PRJNA699172 | JAFDNJ000000000 | Wu et al., 2022 |
| 1679 | Environment | NCBI | PRJNA699172 | JAFDNI000000000 | Wu et al., 2022 |
| 1798 | Dairy | NCBI | PRJNA699172 | JAFDNH000000000 | Wu et al., 2022 |
| 1880 | Vegetables | NCBI | PRJNA699172 | JAFDNG000000000 | Wu et al., 2022 |
| 1976 | Environment | NCBI | PRJNA699172 | JAFDNF000000000 | Wu et al., 2022 |
| 1989 | Seafood | NCBI | PRJNA699172 | JAFDNE000000000 | Wu et al., 2022 |
| 1991 | Seafood | NCBI | PRJNA699172 | JAFDND000000000 | Wu et al., 2022 |
| 2075 | Environment | NCBI | PRJNA699172 | JAFDNC000000000 | Wu et al., 2022 |
| 2095 | Environment | NCBI | PRJNA699172 | JAFDNB000000000 | Wu et al., 2022 |
| 2105 | Environment | NCBI | PRJNA699172 | JAFDNA000000000 | Wu et al., 2022 |
| 2113 | Environment | NCBI | PRJNA699172 | JAFDMZ000000000 | Wu et al., 2022 |
| 2181 | Environment | NCBI | PRJNA699172 | JAFDMY000000000 | Wu et al., 2022 |
| 2183 | Environment | NCBI | PRJNA699172 | JAFDMX000000000 | Wu et al., 2022 |
| 2185 | Environment | NCBI | PRJNA699172 | JAFDMW000000000 | Wu et al., 2022 |
| 2226 | Environment | NCBI | PRJNA699172 | JAFDMV000000000 | Wu et al., 2022 |
| 2234 | Environment | NCBI | PRJNA699172 | JAFDMU000000000 | Wu et al., 2022 |
| 2237 | Environment | NCBI | PRJNA699172 | JAFDMT000000000 | Wu et al., 2022 |
| 2240 | Environment | NCBI | PRJNA699172 | JAFDMS000000000 | Wu et al., 2022 |
| 2241 | Environment | NCBI | PRJNA699172 | JAFDMR000000000 | Wu et al., 2022 |
| 2256 | Environment | NCBI | PRJNA699172 | JAFDMQ000000000 | Wu et al., 2022 |
| 2259 | Environment | NCBI | PRJNA699172 | JAFDMP000000000 | Wu et al., 2022 |
| 2294 | Environment | NCBI | PRJNA699172 | JAFDMO000000000 | Wu et al., 2022 |
| 2298 | Environment | NCBI | PRJNA699172 | JAFDMN000000000 | Wu et al., 2022 |
| 2507 | Environment | NCBI | PRJNA699172 | JAFDMM000000000 | Wu et al., 2022 |
| 3442 | Vegetables | NCBI | PRJNA714047 | JAGEUO000000000 | Wu et al., 2022 |

| | | | | | |
|---|---|---|---|---|---|
| 3443 | Vegetables | NCBI | PRJNA714047 | JAGEUM000000000 | Wu et al., 2022 |
| 3444 | Vegetables | NCBI | PRJNA714047 | JAGEUN000000000 | Wu et al., 2022 |
| 3445 | Vegetables | NCBI | PRJNA714047 | JAGEUL000000000 | Wu et al., 2022 |
| 3446 | Vegetables | NCBI | PRJNA714047 | JAGEUJ000000000 | Wu et al., 2022 |
| 3447 | Vegetables | NCBI | PRJNA714047 | JAGEUK000000000 | Wu et al., 2022 |
| 3448 | Vegetables | NCBI | PRJNA714047 | JAGEUI000000000 | Wu et al., 2022 |
| 1513C | Environment | NCBI | PRJNA698557 | JAFFOM000000000 | Wu et al., 2022 |
| F1109-17 | Environment | NCBI | PRJNA788387 | SAMN2396067 | Wu et al., 2022 |
| F111-17 | Mixed foods | NCBI | PRJNA788387 | SAMN2396068 | Wu et al., 2022 |
| F112-17 | Mixed foods | NCBI | PRJNA788387 | SAMN2396069 | Wu et al., 2022 |
| F114-17 | Mixed foods | NCBI | PRJNA788387 | SAMN2396070 | Wu et al., 2022 |
| F1209-16 | Environment | NCBI | PRJNA788387 | SAMN2396071 | Wu et al., 2022 |
| F1221-16 | Meat | NCBI | PRJNA788387 | SAMN2396072 | Wu et al., 2022 |
| F1230-16 | Meat | NCBI | PRJNA788387 | SAMN2396073 | Wu et al., 2022 |
| F1524-16 | Environment | NCBI | PRJNA788387 | SAMN2396074 | Wu et al., 2022 |
| F1644-17 | Environment | NCBI | PRJNA788387 | SAMN2396075 | Wu et al., 2022 |
| F1646-17 | Environment | NCBI | PRJNA788387 | SAMN2396076 | Wu et al., 2022 |
| F168-17 | Environment | NCBI | PRJNA788387 | SAMN2396077 | Wu et al., 2022 |
| F1857-15 | Environment | NCBI | PRJNA788387 | SAMN2396078 | Wu et al., 2022 |
| F2151-17 | Meat | NCBI | PRJNA788387 | SAMN2396080 | Wu et al., 2022 |
| F2152-17 | Meat | NCBI | PRJNA788387 | SAMN2396081 | Wu et al., 2022 |
| F2153-17 | Meat | NCBI | PRJNA788387 | SAMN2396082 | Wu et al., 2022 |
| F2154-17 | Meat | NCBI | PRJNA788387 | SAMN2396083 | Wu et al., 2022 |
| F2155-17 | Meat | NCBI | PRJNA788387 | SAMN2396084 | Wu et al., 2022 |
| F2160-17 | Meat | NCBI | PRJNA788387 | SAMN2396085 | Wu et al., 2022 |

| | | | | | |
|---|---|---|---|---|---|
| F2161-17 | Environment | NCBI | PRJNA788387 | SAMN2396086 | Wu et al., 2022 |
| F2165-17 | Environment | NCBI | PRJNA788387 | SAMN2396087 | Wu et al., 2022 |
| F2166-17 | Environment | NCBI | PRJNA788387 | SAMN2396088 | Wu et al., 2022 |
| F2170-17 | Environment | NCBI | PRJNA788387 | SAMN2396089 | Wu et al., 2022 |
| F2176-17 | Environment | NCBI | PRJNA788387 | SAMN2396090 | Wu et al., 2022 |
| F2299-15 | Environment | NCBI | PRJNA788387 | SAMN2396091 | Wu et al., 2022 |
| F340-17 | Environment | NCBI | PRJNA788387 | SAMN2396092 | Wu et al., 2022 |
| F345-17 | Environment | NCBI | PRJNA788387 | SAMN2396093 | Wu et al., 2022 |
| F347-16 | Environment | NCBI | PRJNA788387 | SAMN2396094 | Wu et al., 2022 |
| F347-17 | Environment | NCBI | PRJNA788387 | SAMN2396095 | Wu et al., 2022 |
| F348-17 | Environment | NCBI | PRJNA788387 | SAMN2396096 | Wu et al., 2022 |
| F365-16 | Meat | NCBI | PRJNA788387 | SAMN2396097 | Wu et al., 2022 |
| F671-16 | Environment | NCBI | PRJNA788387 | SAMN2396098 | Wu et al., 2022 |
| F742-17 | Environment | NCBI | PRJNA788387 | SAMN2396099 | Wu et al., 2022 |
| F991-16 | Environment | NCBI | PRJNA788387 | SAMN2396100 | Wu et al., 2022 |
| MQ130026 | Clinical | NCBI | PRJNA371539 | MUZG00000000 | Hilliard et al., 2018 |
| MQ130029 | Clinical | NCBI | PRJNA371539 | MVED00000000 | Hilliard et al., 2018 |
| MQ130032 | Clinical | NCBI | PRJNA371539 | MVEE00000000 | Hilliard et al., 2018 |
| MQ130033 | Clinical | NCBI | PRJNA371539 | MVEF00000000 | Hilliard et al., 2018 |
| MQ130037 | Clinical | NCBI | PRJNA371539 | MVFA00000000 | Hilliard et al., 2018 |
| MQ130042 | Clinical | NCBI | PRJNA371539 | MVEG00000000 | Hilliard et al., 2018 |
| MQ130058 | Clinical | NCBI | PRJNA371539 | MVEH00000000 | Hilliard et al., 2018 |
| MQ140011 | Clinical | NCBI | PRJNA371539 | MVEI00000000 | Hilliard et al., 2018 |
| MQ140012 | Clinical | NCBI | PRJNA371539 | MVEJ00000000 | Hilliard et al., 2018 |
| MQ140025 | Clinical | NCBI | PRJNA371539 | MVEK00000000 | Hilliard et al., 2018 |

| | | | | | |
|---|---|---|---|---|---|
| MQ140029 | Clinical | NCBI | PRJNA371539 | MVEL00000000 | Hilliard et al., 2018 |
| MQ140030 | Clinical | NCBI | PRJNA371539 | MVEM00000000 | Hilliard et al., 2018 |
| MQ140031 | Clinical | NCBI | PRJNA371539 | MVEN00000000 | Hilliard et al., 2018 |
| MQ140032 | Clinical | NCBI | PRJNA371539 | MVEO00000000 | Hilliard et al., 2018 |
| MQ140033 | Clinical | NCBI | PRJNA371539 | MVEP00000000 | Hilliard et al., 2018 |
| MQ140034 | Clinical | NCBI | PRJNA371539 | MVEQ00000000 | Hilliard et al., 2018 |
| MQ140035 | Clinical | NCBI | PRJNA371539 | MVER00000000 | Hilliard et al., 2018 |
| MQ150001 | Clinical | NCBI | PRJNA371539 | MVES00000000 | Hilliard et al., 2018 |
| MQ150004 | Clinical | NCBI | PRJNA371539 | MVET00000000 | Hilliard et al., 2018 |
| MQ150005 | Clinical | NCBI | PRJNA371539 | MVEU00000000 | Hilliard et al., 2018 |
| MQ150007 | Clinical | NCBI | PRJNA371539 | MVEV00000000 | Hilliard et al., 2018 |
| MQ150008 | Clinical | NCBI | PRJNA371539 | MVEW00000000 | Hilliard et al., 2018 |
| MQ150011 | Clinical | NCBI | PRJNA371539 | MVEX00000000 | Hilliard et al., 2018 |
| MQ150012 | Clinical | NCBI | PRJNA371539 | MVEY00000000 | Hilliard et al., 2018 |
| MQ150013 | Clinical | NCBI | PRJNA371539 | MVEZ00000000 | Hilliard et al., 2018 |
| G-025 | Clinical | ENA | PRJEB88151 | ERS23972866 | This study |
| G-026 | Clinical | ENA | PRJEB88151 | ERS23972867 | This study |
| G-028 | Clinical | ENA | PRJEB88151 | ERS23972869 | This study |
| G-030 | Clinical | ENA | PRJEB88151 | ERS23972870 | This study |
| G-031 | Clinical | ENA | PRJEB88151 | ERS23972871 | This study |
| G-033 | Clinical | ENA | PRJEB88151 | ERS23972873 | This study |
| G-034 | Clinical | ENA | PRJEB88151 | ERS23972874 | This study |
| G-035 | Clinical | ENA | PRJEB88151 | ERS23972875 | This study |
| G-037 | Clinical | ENA | PRJEB88151 | ERS23972877 | This study |
| G-038 | Clinical | ENA | PRJEB88151 | ERS23972878 | This study |
| G-039 | Clinical | ENA | PRJEB88151 | ERS23972880 | This study |
| G-040 | Clinical | ENA | PRJEB88151 | ERS23972881 | This study |
| G-041 | Clinical | ENA | PRJEB88151 | ERS23972882 | This study |
| G-043 | Clinical | ENA | PRJEB88151 | ERS23972884 | This study |
| G-045 | Clinical | ENA | PRJEB88151 | ERS23972885 | This study |
| G-046 | Clinical | ENA | PRJEB88151 | ERS23972887 | This study |
| G-048 | Clinical | ENA | PRJEB88151 | ERS23972889 | This study |
| G-002 | Clinical | ENA | PRJEB26050 | ERR2521731 | This study |

| | | | | | |
|---|---|---|---|---|---|
| G-003 | Clinical | ENA | PRJEB26050 | ERR2521734 | This study |
| G-004 | Clinical | ENA | PRJEB26050 | ERR2521733 | This study |
| G-005 | Clinical | ENA | PRJEB26050 | ERR2521735 | This study |
| G-006 | Clinical | ENA | PRJEB26050 | ERR2521736 | This study |
| G-007 | Clinical | ENA | PRJEB26050 | ERR2521737 | This study |
| G-008 | Clinical | ENA | PRJEB26050 | ERR2521738 | This study |
| G-010 | Clinical | ENA | PRJEB26050 | ERR2521740 | This study |
| G-011 | Clinical | ENA | PRJEB26050 | ERR2521742 | This study |
| G-012 | Clinical | ENA | PRJEB26050 | ERR2521743 | This study |
| G-013 | Clinical | ENA | PRJEB26050 | ERR2521744 | This study |
| G-014 | Clinical | ENA | PRJEB26050 | ERR2521745 | This study |
| G-015 | Clinical | ENA | PRJEB26050 | ERR2521746 | This study |
| G-016 | Clinical | ENA | PRJEB26050 | ERR2521747 | This study |
| G-017 | Clinical | ENA | PRJEB26050 | ERR2521741 | This study |
| G-018 | Clinical | ENA | PRJEB26050 | ERR2521751 | This study |
| G-020 | Clinical | ENA | PRJEB26050 | ERR2521761 | This study |
| G-021 | Clinical | ENA | PRJEB26050 | ERR2521749 | This study |
| G-022 | Clinical | ENA | PRJEB26050 | ERR2521758 | This study |
| G-023 | Clinical | ENA | PRJEB26050 | ERR2521756 | This study |
| G-024 | Clinical | ENA | PRJEB26050 | ERR2521754 | This study |
| EGD-e | | NCBI | PRJNA276 | SAMEA3138329 | |
| 10403S | | NCBI | PRJNA17495 | SAMN02641382 | |
| 6179 | | NCBI | PRJEB1355 | SAMEA3138937 | |
| F2365 | | NCBI | PRJNA590944 | SAMN13941796 | |
